# Prophylactic use of vancomycin powder on postoperative infection after total joint arthroplasty

**DOI:** 10.1186/s12891-023-07024-2

**Published:** 2024-01-16

**Authors:** Jian Gao, Li Shu, Kan Jiang, Aikeremujiang Muheremu

**Affiliations:** Orthopedic Research Center, Sixth Affiliated Hospital of Xinjiang Medical University, 39, Wuxing Nan Rd, Tianshan District, 86830001, 86830001 Urumqi, Xinjiang China

**Keywords:** TJA, Vancomycin, Local use, Infected, Prevention, Meta-analysis

## Abstract

**Objective:**

By reviewing the literature analyzing vancomycin powder for preventive surgery, the effect of this method on reducing the infection rate after TJA was systematically evaluated to provide a basis for future clinical work.

**Methods:**

Using PubMed, Medline, Elsevier, and CNKI, with the following mesh words: “vancomycin”, “local / intraoperative / topical / intrawound”, “TJA”, “TKA”, “THA”, “total joint arthroplasty”, “total knee arthroplasty”, “total hip arthroplasty”, “infection”, and “SSI”, to search for case-control research papers on the impact of prophylactic application of vancomycin powder on the incidence of postoperative infection, we compared the overall infection rate in the literature by using RevMan 5.3 meta-analysis software and analyzed the impact of vancomycin on the infection rate of different parts and types of TJA according to different subgroups.

**Results:**

A total of 22 qualified studies were selected; twenty-five studies compared the effect of prophylactic use of vancomycin powder on infection rates after TJA. There were 23,363 cases in total, including 9545 cases in the vancomycin group and 13,818 cases in the control group. The results of the meta-analysis showed that the possibility of postoperative infection after prophylactic use of vancomycin powder was significantly lower than that without vancomycin risk ratio: 0.38 [0.23,0.59], P < 0.01). However, a meta-analysis of randomized controlled trials (RCTs) showed no significant effect of vancomycin on postoperative infection (P = 0.52).

**Conclusion:**

Based on the retrospective studies, local prophylactic use of vancomycin powder in TJA can significantly reduce the incidence of postoperative infection. High-quality RCTs should be carried out to further evaluate these results.

Although aseptic technology in total joint replacement (TJA) has made great progress, the occurrence of postoperative surgical site infection (SSI) infection cannot be avoided in many cases. In the current report, the infection rate after TJA was between 2% and 13% [1, 2]. In the United States, there are approximately half a million cases of SSI every year, resulting in a direct economic loss of US $1.8 billion [3]. SSI brings great physical, psychological and economic pressure to patients. The hospital stay of patients caused by postoperative infection is prolonged by up to two weeks, and patients must face the risk of a higher cost of treatment, a higher disability rate and a higher mortality rate [4].

At present, infection control has become an important evaluation standard for the level of diagnosis and treatment in medical centers. A variety of techniques have been used to control postoperative infection, such as careful skin preparation and disinfection of the operation field, timely administration of appropriate antibiotics, timely isolation of patients carried by MRSA, massive intraoperative flushing and early removal of drainage tube after operation [5, 6]. A variety of antibiotics have been locally used to prevent infection after orthopedic surgery since the 1970s [7, 8]. Considering that the microorganisms leading to postoperative infection are mainly Staphylococcus aureus and Staphylococcus epidermidis, local application of vancomycin in the operation site may be an effective method to prevent postoperative infection of joints [9]. In recent years, vancomycin powder has been used in many orthopedic centers, and many have reported satisfactory results. However, there is no unified conclusion on whether this method can reduce the postoperative infection rate. The current study analyzed the relevant literature on the comparison of the incidence of infection after TJA with or without vancomycin powder, verified the feasibility of this method to prevent infection, and provided an evidence-based medical basis for the clinical practice of TJA.

## Data and methods

### Literature review

Two researchers independently searched MEDLINE (1990–2023), PubMed (1990–2023), Elsevier (1990–2023), EMBASE (1990–2023) and Cochrane Library (2008–2023) using the keywords “vancomycin”, “local / intraoperative / topical / intrawound”, “total joint arthroplasty/ total knee arthroplasty / total hip arthroplasty”, “TJA”, “TKA”, “THA”, “infection”, and “SSI” for studies about vancomycin-based SSI prevention after arthroplasty. The PROSPERO registration number of the study protocol is 314,732. This meta-analysis of the data from published studies thus requires no institutional review board (IRB) approval.

### Inclusion and exclusion criteria

Inclusion criteria: All retrospective and prospective studies on the topical use of vancomycin to prevent infection after TJA published before April 2023.

Exclusion criteria: Case reports, reviews, expert opinions and lectures were excluded.

### Literature quality evaluation

Two authors independently evaluated the quality of the selected literature using the Newcastle Ottawa Scale (NOS) scoring standard [10]. The risks of bias in the included studies were assessed using the Cochrane Risk of Bias Tool (Review Manager 5.4). The items assessed were (1) random sequence generation (selection bias), (2) allocation concealment (selection bias), (3) blinding of participants and personnel (performance bias), (4) blinding of outcome assessment (detection bias), (5) incomplete outcome data (attrition bias), (6) selective reporting (reporting bias) and (7) other biases.

### Outcome indicators

The rate of infection after TJA with or without topical vancomycin powder.

### Statistical analysis

We used Cochrane Collaboration Network’s RevMan5.3 to analyze the extracted data. The heterogeneity of the included studies was assessed by the chi-square test, and heterogeneity was judged by I^2^. The random effect model was used due to the difference of included patient characteristics from different studies.

## Results

### Original data analysis

**Fig. 1:**
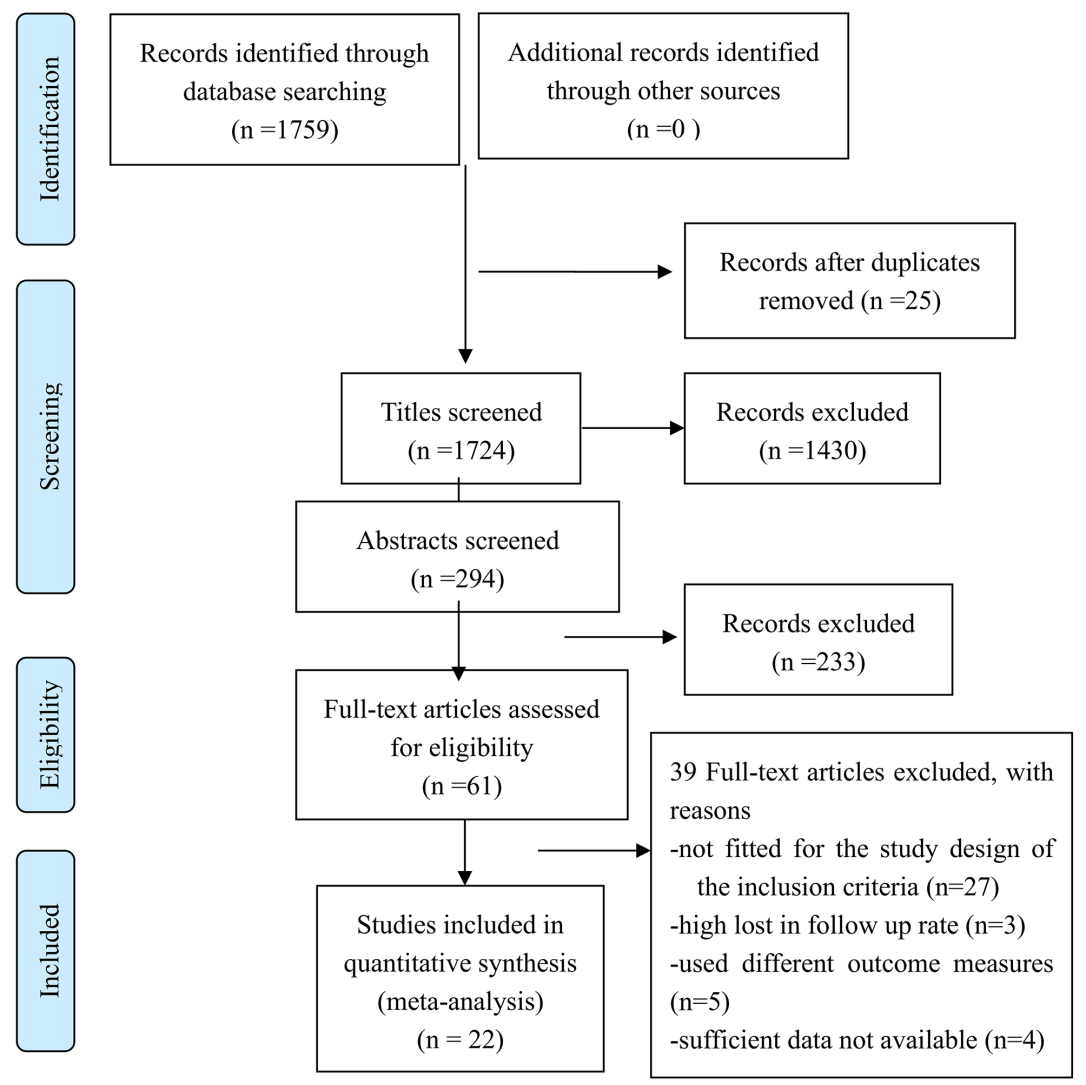
Flow chart of the study inclusion process

A total of 1759 papers were found, and 22 qualified studies were selected for final analysis [11–32]. There were 23,363 cases in total, including 9545 cases in the preventive vancomycin group and 13,818 cases in the control group. The process is shown in Fig. 1; Table 1.

**Table 1:** demographic characteristics of the included studies

Author ( Year)	Patients Size	Study design	Level of Evidence	Quality (RoB)	Dose (grams)	Follow up (months)	Main outcome
Abuzaiter 2023	165	RCT	II	5	1	12	Incidence of SSI
Aljuhani 2021	96	Retrospective	III	6	2	24	Incidence of SSI
Assor 2009	135	Retrospective	III	6	1	24	Incidence of SSI
Cohen A 2018	1502	Retrospective	III	7	1	24	Incidence of SSI
Cohen E 2019	555	Retrospective	III	7	1	18	Incidence of SSI
Crawford D2018	1885	Retrospective	III	6	1	12	Incidence of SSI
Dial 2018	265	Retrospective	III	6	1	8	Incidence of SSI
Duan 2022	2725	Retrospective	III	7	2	3	Incidence of SSI
Erken 2020	93	Retrospective	III	7	1	12	Incidence of SSI
Hanada 2019	166	Retrospective	III	6	1	12	Incidence of SSI
Khatri 2017	115	Retrospective	III	6	1	6	Incidence of SSI
Koutalos 2020	290	Retrospective	III	7	2	24	Incidence of SSI
Matziolis2020	8945	Retrospective	III	6	1	12	Incidence of SSI
Otte 2017	1640	Retrospective	III	7	1	12	Incidence of SSI
Patel 2018	460	Retrospective	III	6	1	12	Incidence of SSI
Riesgo 2018	74	Retrospective	III	7	1	12	Incidence of SSI
Tahmaseb 2021	2024	Retrospective	II	7	1	12	Incidence of SSI
Wang 2023	90	RCT	II	3	0.5-1	3	Incidence of SSI
Winkler 2018	744	Retrospective	III	6	2	24	Incidence of SSI
Wu 2022	90	RCT	II	3	0.5-1	3	Incidence of SSI
Xu 2020	855	Retrospective	III	6	1	12	Incidence of SSI
Yavuz 2019	976	Retrospective	III	6	1	24	Incidence of SSI

Among the 22 studies finally included in this study, 2 were prospective randomized controlled studies, and the other 23 were retrospective studies. These studies were published between 2011 and 2023. The level of evidence was between 2 and 3.

### Comparison of overall Infection rate

**Fig. 2:**
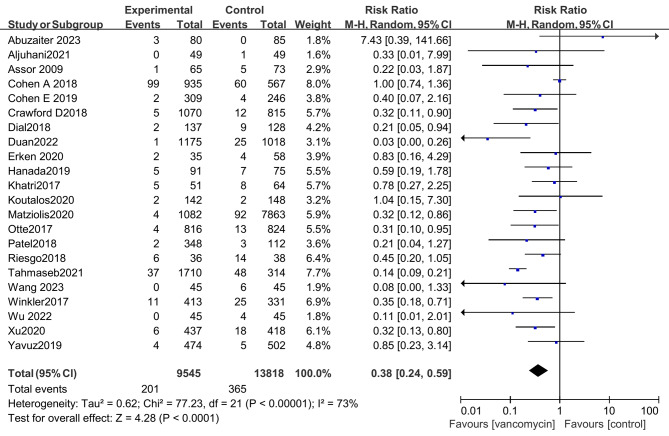
The results of the overall meta-analysis showed that the possibility of postoperative infection after prophylactic use of vancomycin powder was significantly lower than that in the control group

Twenty-two studies compared the effect of prophylactic use of vancomycin powder on infection rates after TJA since I^2^ > 50%, and the meta-analysis used a random effect model. The results showed that the possibility of postoperative infection after prophylactic use of vancomycin powder was significantly lower than that in the control group (risk ratio: 0.38 [0.24, 0.59], P < 0.01) (Fig. 2).

### Comparison of infection rates concerning primary and revision TKA and THA

**Fig. 3:**
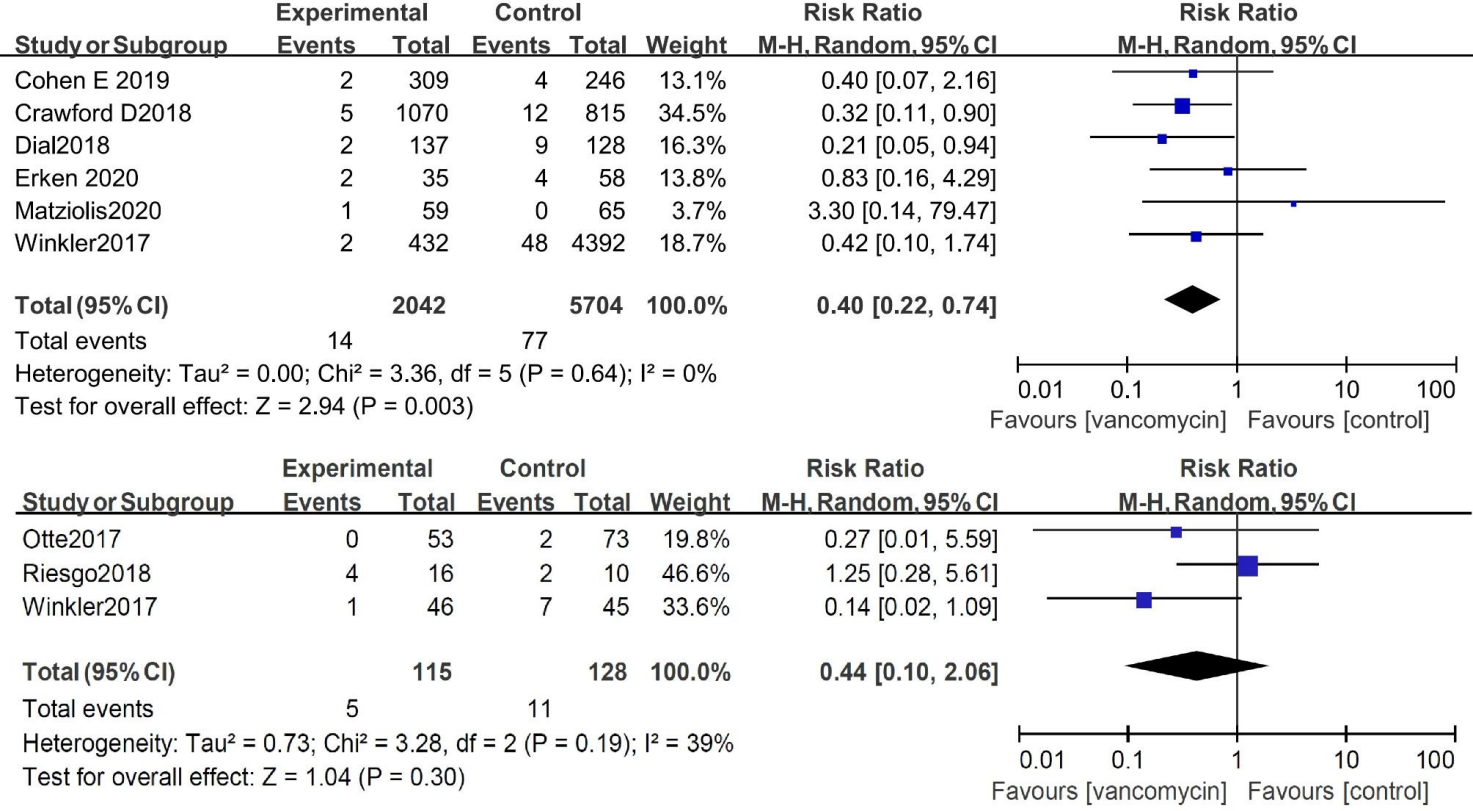
Comparison of the infection rate concerning THA showed that the incidence of SSI after primary or revision THA can be significantly reduced after topical application of vancomycin

**Fig. 4:**
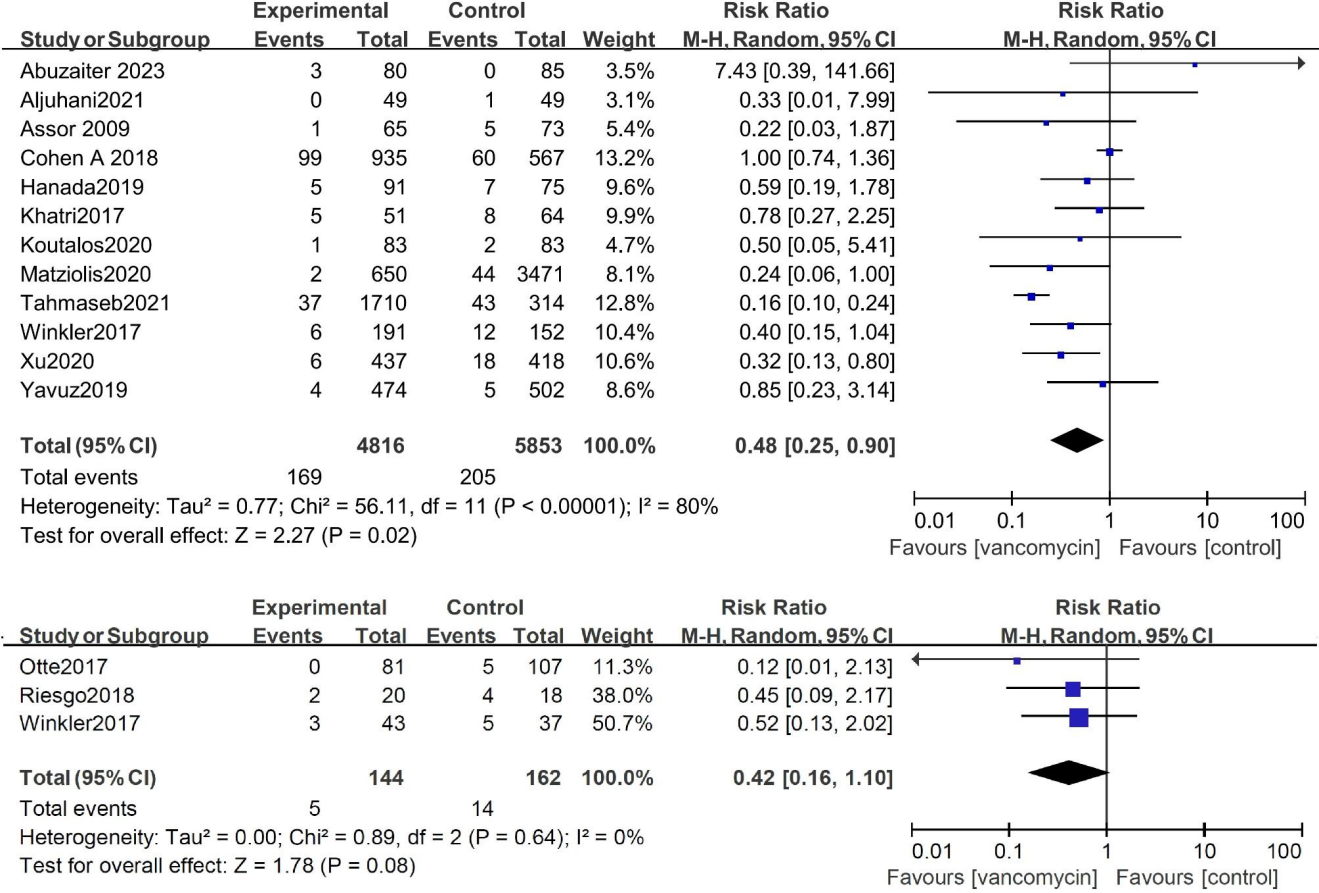
Comparison of the infection rate concerning primary and revision TKA showed that the incidence of SSI after primary THA can be significantly reduced after topical application of vancomycin, but the difference was not significant after revision TKA.

Considering that there could be differences between the infection rates after primary or secondary TKA and THA, we separately compared the infection rates concerning primary and revision TKA and THA after using topical vancomycin. The results of the meta-analysis of 6 studies with 7746 patients showed that the incidence of postoperative infection after primary THA can be significantly reduced after topical application of vancomycin (risk ratio: 0.40 [0.22, 0.74], P < 0.01). However, three studies with 243 patients showed no significant reduction of SSI after topical application of vancomycin in revision THAs (risk ratio: 0.42 [0.16, 1.10], P = 0.08) (Fig. 3). A meta-analysis of 12 studies with 10,669 patients showed that the incidence of postoperative infection after primary TKA can be significantly reduced after topical application of vancomycin (risk ratio: 0.48 [0.25, 0.90], P = 0.02). In addition, three studies with 243 patients showed no significant reduction in infection upon applying topical vancomycin in revision TKA (risk ratio: 0.44 [0.10, 2.06], P = 0.30) (Fig. 4).

### Effect of Vancomycin on deep Infection and superficial Infection

**Fig. 5:**
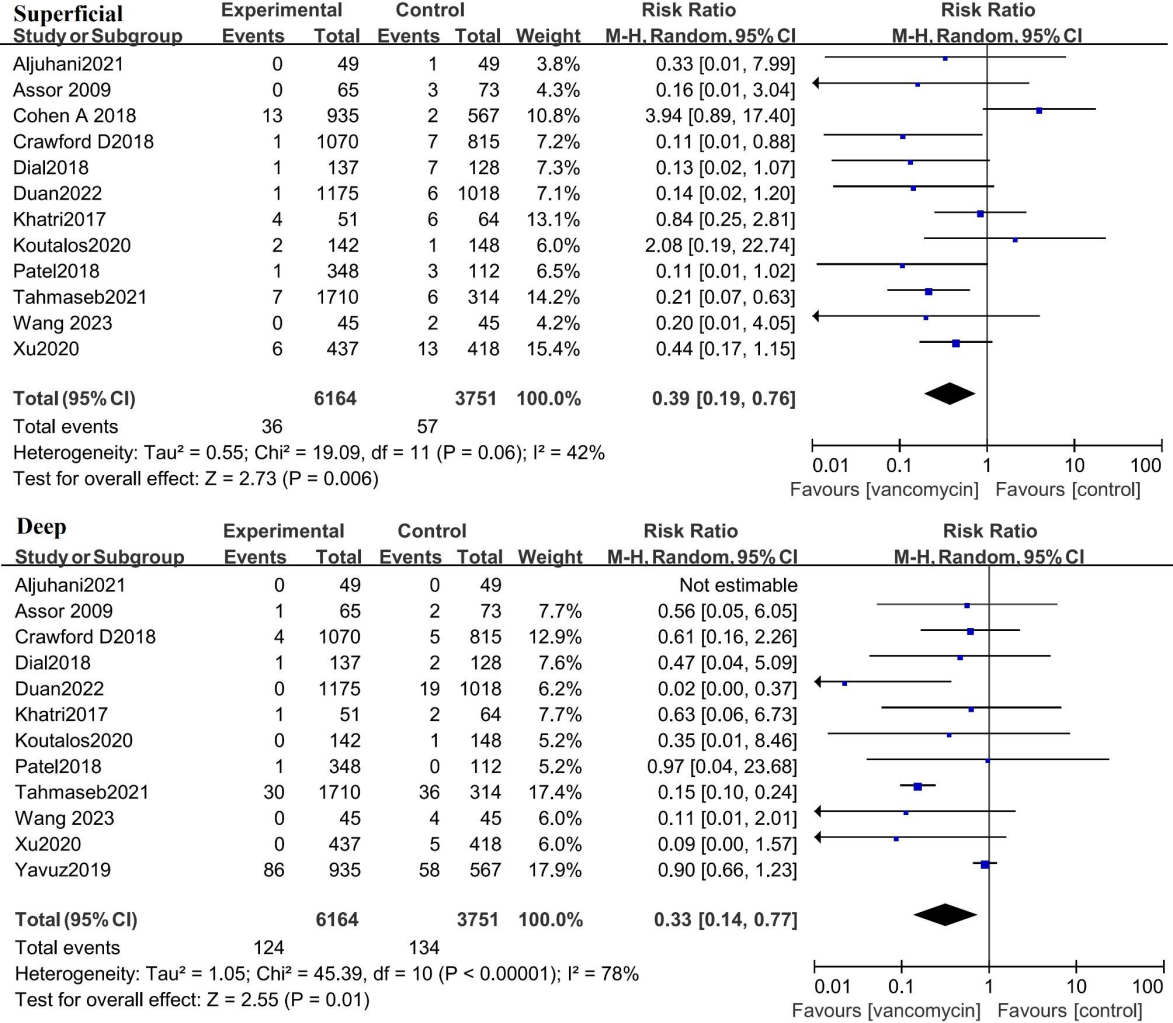
The incidence of both superficial and deep tissue infection after using vancomycin in TJA was significantly lower than that without vancomycin

Considering the different possible adverse consequences and different treatment methods of deep tissue infection and superficial tissue infection, this study analyzed the effects of preventive use of vancomycin on deep and superficial infection. Twelve studies containing data from 9915 patients analyzed the effect of prophylactic use of vancomycin powder on the incidence of superficial tissue infection. The results of the meta-analysis showed that the incidence of superficial tissue infection after using vancomycin in TJA was significantly lower than that without vancomycin (risk ratio: 0.39 [0.19, 0.76], P < 0.01) (Fig. 5). In addition, the incidence of deep tissue infection after using vancomycin in TJA was also significantly lower than that without vancomycin (risk ratio: 0.33 [0.14, 0.77], P = 0.01) (Fig. 5).

### Analysis of randomized controlled trials and retrospective studies

**Fig. 6:**
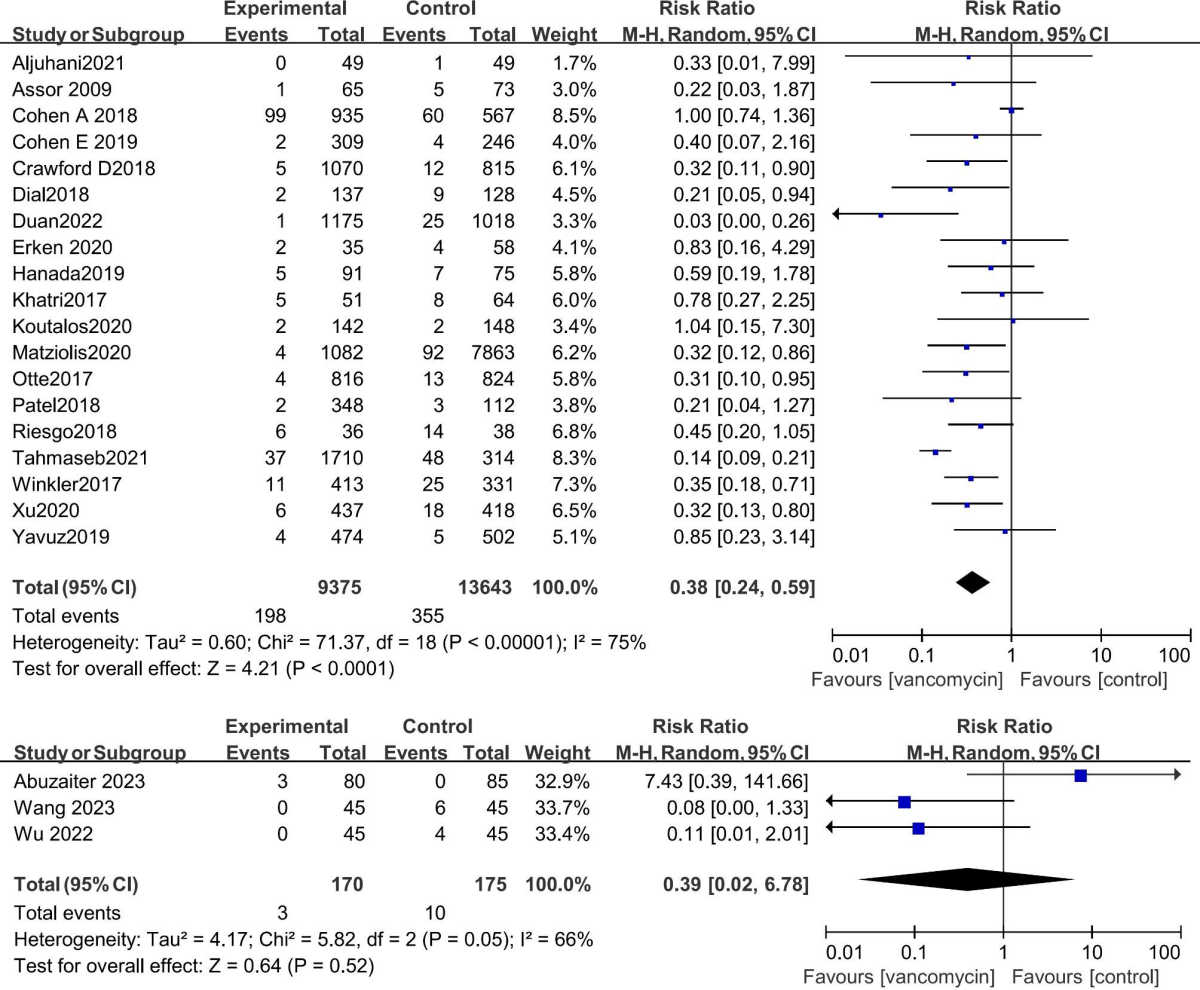
Meta-analysis of retrospective studies showed that the incidence of SSI after TJA can be significantly reduced after topical application of vancomycin, while no such significant difference was found from the meta-analysis of RCTs.

Considering the advantages of randomized controlled studies over retrospective studies, we analyzed the two types of studies. At present, there are three randomized controlled studies on the prophylactic use of vancomycin powder in TJA, and the results show that this method has no significant effect on the incidence of postoperative infection. The data of 345 patients were included in the three studies (risk ratio: 0.39 [0.02, 6.78], P = 0.52) (Fig. 6). The results of 19 retrospective studies including 23,018 patients showed that the infection rate after using vancomycin in TJA was significantly lower than that without vancomycin (risk ratio: 0.36 [0.22, 0.59], P < 0.01) (Fig. 6). However, the three randomized controlled studies did not provide the registration number of the randomized controlled study and were completed in a single treatment center, compromising the reliability of their results. More randomized controlled studies are needed for further meta-analysis.

## Discussion

The incidence of SSI, a leading cause of revision in TJA, has been reported to range between 1% and 4% after primary TKA and 1–2% after primary THA [33]. Obesity, diabetes, advanced age, poor or general health, smoking, prolonged operations, and substantial blood loss are risk factors for a high ratio of SSI [34]. The number of revisions increases in proportion to the number of primary implants performed each year, with a projected 176% increase in revision between 2014 and 2030 in THA and a 170% increase in TKA [35]. Patients with SSI have a significantly higher mortality rate than those undergoing aseptic revisions, up to five times higher at one year [36]. Economic studies have estimated that the annual cost associated with SSI after TJA in the United States was approximately one billion dollars in 2017 and is projected to reach nearly two billion dollars by 2030 [37].

The topical use of this antibiotic has been widely adopted in various fields of orthopedic surgery, and it has shown promising results. While topical application of vancomycin can obtain a high drug concentration at the surgical site and minimize the potential harm of high-dose vancomycin to healthy organs, intravenous injection of vancomycin may cause certain serious adverse responses, such as impairment of liver and kidney function. Rarely are there reports of permanent harm brought on by topical vancomycin treatment in the present literature [38]. In our previous meta-analysis, we found that the application of VP powder before closing the wound resulted in a significant decrease in postoperative deep wound infection rates in spinal fusions with posterior instrumentation [39]. Another study, a multicentre randomized clinical trial, reported that the application of 1 g of VP was linked to a reduced risk of deep surgical site infection in tibial fractures caused by gram-positive organisms [40]. Furthermore, the use of VP in shoulder arthroplasty procedures was associated with a significant decrease in prosthetic joint infection (PJI) without an increased rate of non-infectious wound complications [41]. Considering these positive outcomes, VP is being employed in total joint arthroplasty (TJA) with the goal of significantly lowering the risk of PJI. The topical application of VP allows for higher concentrations in the surgical area while minimizing systemic side effects. In a rat model, the use of intra-articular VP combined with intravenous antibiotics resulted in complete eradication of MRSA bacteria from contaminated implants [42].

However, although numerous studies have produced excellent results, the data from these investigations differ. Here, we examine a total of 22 trials including 23,363 individuals to assess the effectiveness of topical vancomycin powder as a preventative measure during TJA. Consequently, it is possible that selection bias may exist. According to our findings, a local vancomycin supply can significantly reduce the risk of SSI following TJA (risk ratio: 0.38 [0.24, 0.59], P < 0.01), indicating a clear advantage for using topical vancomycin powder during total joint replacement procedures.

Since the essence of included patient characteristics from different studies are quite different (ex. Age, race, TJA procedure protocol), here we used a random effect model to avoid selection bias in the meta-analysis. Considering the possible differences in the ratio of SSI after different surgical approaches, we also analyzed the incidence of SSI after different surgical scenarios. These include superficial or deep tissue infections after primary or revision THA and TKA. The results showed that local use of vancomycin could significantly reduce the incidence of SSI after primary and revision THA as well as primary TKA. Although no significant difference was found when comparing the incidence of SSI after revision TKA, significant results could be achieved when more studies with revision TKA were included in the meta-analysis.

Among the 22 studies that were a part of this systematic review, 19 were retrospective case-control studies, and three were randomized controlled studies. All the papers were written in English. The included studies have an evidence level of two to three, and the majority of them have quality scores (NOS) of six to eight. Aside from all sharing the limitation of using mostly level II and III studies, they also tend to include confounding variables, which might result in biased conclusions. As a result, even with numerous recently published studies, we still lack essential knowledge about the effectiveness of VP in reducing PJI in TJA. To address this problem, it would be crucial to conduct a randomized controlled trial that follows a consistent methodology and excludes any additional confounding variables.

There are three RCTs so far published on this topic, and the meta-analysis of those three studies is not consistent with the meta-analysis of non-RCT studies. However, we believe that the results of the meta-analysis of RCTs are not reliable. First, although the two papers published by Wu and Wang et al. were the results of two different studies in two different affiliations, the data on the number of patients, patient age, gender, and method of treatment were exactly the same. In the results section, the body temperature, neutrophil count, IL-6 and CRP were exactly the same. Therefore, we are doubtful about the authenticity of those numbers and do not recommend using them in future meta-analyses. Then, in the RCT of Abuzaiter et al., apart from the standard preoperative IV antibiotics (2 to 3 g of cefazolin) within 60 min before skin incision, patients in the control group continued the aforementioned IV antibiotics every 8 h for 2 doses postoperatively, while those randomized to the treatment group did not receive standard postoperative antibiotics but rather were administered 1 g of topical VP, applied intraoperatively by the orthopedic surgeon (500 mg directly around the prosthesis and 500 mg above the closed joint capsule) before wound closure. It is possible that the low dosage of intraoperative VP and the absence of postoperative IV antibiotics could be the reason for the different results of this RCT from the meta-analysis of non-RCT studies.

Although there are some meta-analyses published on this topic, the current paper is the most comprehensive analysis of the current literature with the largest inclusion of studies and patients. However, while proving the applicability of vancomycin powder after TJA in reducing SSI, our study has certain limitations. Most of the included studies were retrospective studies, and the statistical efficacy of meta-analysis needs to be further improved by including better designed RCTs. The duration of follow-up time varied among studies, but none were longer than 24 months. The results of the studies could change if randomization and a longer follow-up time are applied. Moreover, due to the difference in hardware and the experience of different surgeons, the results of the studies could vary for reasons other than the application of vancomycin. In the future, meta-analyses based on more high-quality RCTs can overcome the above shortcomings.

## Conclusion

Through the meta-analysis of previous retrospective clinical case-control studies, our study suggests that prophylactic application of vancomycin powder can significantly reduce the incidence of SSI after TJA. However, the statistical efficacy of meta-analysis needs to be further improved by including high-quality RCTs with larger patient sizes.

## Data Availability

Datasets can be accessed from the corresponding author upon request.
